# Luscus: molecular viewer and editor for MOLCAS

**DOI:** 10.1186/s13321-015-0060-z

**Published:** 2015-04-29

**Authors:** Goran Kovačević, Valera Veryazov

**Affiliations:** Division of Materials Physics, Ruđer Bošković Institute, Bijenička 54, P.O.Box 180, Zagreb, HR-10002 Croatia; Theoretical Chemistry, P.O.B. 124, Lund University, Lund, 22100 Sweden

**Keywords:** Molecular modelling, Software, Graphics, Molecular editor, Visualisation

## Abstract

The novel program for graphical display and editing of molecular systems, *luscus*, is described. The program allows fast and easy building and/or editing different molecular structures, up to several thousands of atoms large. *Luscus* is able to visualise dipole moments, normal modes, molecular orbitals, electron densities and electrostatic potentials. In addition, simple geometrical objects can be rendered in order to reveal a geometrical feature or a physical quantity. The program is developed as a graphical interface for the MOLCAS program package, however its adaptive nature makes possible to use *luscus* with other computational program packages and chemical formats. All data files are opened via simple plug-ins which makes easy to implement a new file format in *luscus*. The easiness of editing molecular geometries makes *luscus* suitable for teaching students chemical concepts and molecular modelling.

**Electronic supplementary material:**

The online version of this article (doi:10.1186/s13321-015-0060-z) contains supplementary material, which is available to authorized users.

**Graphical Abstract:**
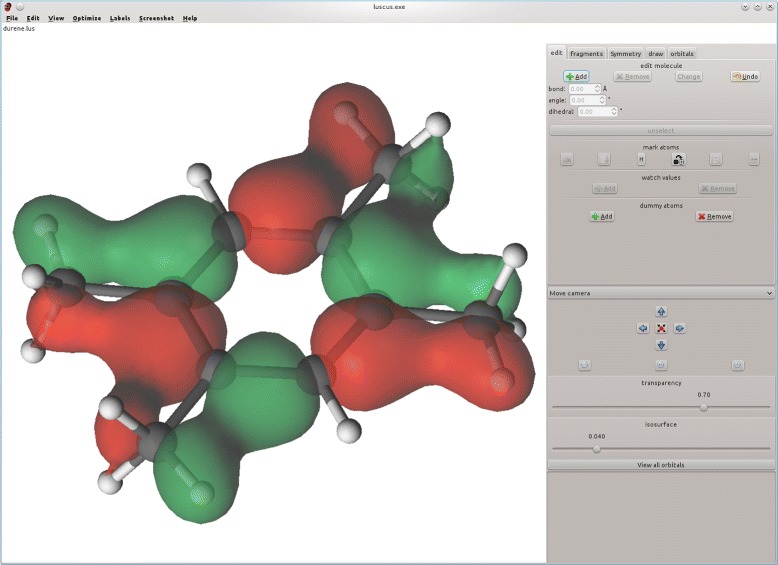
Screenshot of luscus program showing molecular orbital.

## Background

Visualisation of various data in theoretical chemistry plays an important role in the conduction of modern scientific research in the field. Computational possibilities of modern software routinely used in the quantum chemistry and the molecular mechanics and dynamics (MM/MD) can handle very large molecular systems containing hundreds or thousands of atoms. Without advanced graphical tools, designed for a specific branch of computational codes, it is very complicated task to create an input and proceed with calculations.

Although the majority of graphical user interface (GUI) programs used in quantum chemistry have a focus on the preparation of initial molecular geometry, the post processing and visualisation of the results is also extremely important. This is especially the case if the study can not be performed as a “black box” calculation. Many advanced computational techniques, for example, multiconfigurational methods in quantum chemistry [1], require intermediate steps, with verification and altering intermediate results to be reused in the following up calculations.

Graphical user interfaces used in quantum chemistry (and in MM/MD simulations) can be classified as a general tools (not connected to any particular code), or specific to some particular computational code. The first group includes molden [2], rasmol [3], avogadro [4] and many others. These graphical programs can perform visualisation of coordinates and some computed properties. The clear advantage of these projects is a generic interface, and thus, an easy learning curve for the novice users. However, the integration level between general purpose GUI and the computational codes is usually very restricted. Another group includes GUI developed exclusively for specific computational software. It is usually the case for the commercial software: GaussView [5], TmoleX [6], Spartan [7] and others. Although a graphical package, which is tightly bound to a computational code, has an obvious advantage, the end user might find inconvenient the necessity to learn a separate interface for any new code.

*Luscus* is a novel program for molecular modelling and analysis of results from quantum-chemical program packages. The graphical interface is designed and is written to match computational package, MOLCAS [8,9], which includes a set of computational codes for various quantum chemical calculations from Hartree-Fock (HF) and Density Functional Theory (DFT) to the multiconfigurational theory and the coupled cluster theory. By design *luscus* communicates with a computational code via transparent and simple interface allowing easy integration with other programs as well, which makes it a universal tool for computational chemists. *Luscus* uses external plug-ins for reading and writing files. These plug-ins are usually simple programs (or scripts) which are performing simple tasks of converting data from one format to another. This technology increases the flexibility, since implementing a new format for a file with chemistry related data can be done without changing of GUI, but by adding or modifying of the corresponding plug-in.

Another consideration we used in the design of *luscus* code is the portability and the minimal dependence on external graphical libraries. It leads to a light-weighted graphical program which can be easily installed on virtually any platform.

*Luscus* is easy to learn and easy to use program. It is based on GV [10,11] code, which was the main GUI for MOLCAS during the last decade. GV has only primitive user interface provided by OpenGL/glut library. Luscus keeps all capabilities of GV and includes new features and improvements, but with more friendly and intuitive interface.

## Implementation

User interface is created with the gtk+ library, and 3D visual display is made with the OpenGL programming library. These libraries have become widespread, on many architectures, and this commonness ensures the portability of *luscus* code on all common computer platforms and operating systems. These libraries make possible interaction with the molecular model displayed on the screen.

There are various file formats that become the standard in different areas in chemistry, for instance PDB file format for biomolecules and CIF file format for crystallographic data. In computational chemistry, in contrary, the standardisation of data formats is still far away from the completion [12]. The most successful attempt to design a standard format in computational chemistry is based on introduction of Chemical Markup Language (XML/CML) [13,14]. However, until now, very few computational codes accepted these kind of file formats both as an user input and as an intermediate files.

The most widespread file format in computational chemistry is so called XYZ file, which is used for the definition of atomic Cartesian coordinates. This file format has a limited usability since it can store only trivial information about molecular geometry. The format is lacking the support of dummy atoms, environment (e.g. atomic charges), and other molecular properties.

We introduced a new file format for interaction between *luscus* and computational software, which is based on XML mark-up. These files can either be produced directly by the MOLCAS code, or converted from the MOLCAS output files using corresponding plug-ins in luscus.

The main purpose for introducing new file format is the transfer and the storage of visualisable data from MOLCAS[8,9] in a standard fashion. The *luscus* program interprets the data blocks in the file and renders them on a screen. The new format was created as a simple, human readable and editable file. The *luscus* file format follows XML/CML format[13-16] where data are structured in sections, where each section contains a well defined set of data. All sections in a file produce a graphical element on a screen, thus *luscus* file format can be regarded as a XML extension to the graphical representation of chemical data. The file header, however is an exception and is identical to the XYZ file. This makes a great advantage since XYZ file format is the most accepted chemical file format in molecular graphics programs, so those programs can interpret the initial portion of the *luscus* file as XYZ file. The another advantage of this solution is that any XYZ file is a valid luscus file (lacking definition of additional graphical elements).

An example of *luscus* file format is given in supplement [see Additional file 1].

Although *luscus* is designed as a graphical interface for the MOLCAS program package, computational codes with other chemical formats can be interpreted by *luscus* after they are converted by the corresponding external plug-in. When a file is opened, *luscus* executes a plug-in, waits until it converts the file to the *luscus* format and opens it. Information about all available plug-ins (file format name, plugin name, extension) is stored in a configuration file and can be customised by the user. Luscus can automatically determine file type according to the data in the configuration file or alternatively, the file type can be specified by the user. The process of the conversion of file formats is done automatically without interference with user. An example of plug-in script that uses Openbabel [17] for opening PDB file is given in supplement [see Additional file 2].

Plug-ins can be used for saving files as well. In the latter case, plug-in converts *luscus* file to the custom file format. The third kind of a plug-in uses the data stored in a *luscus* file, create an input for a computational code, run the calculation, and finally transform the output back to the *luscus* format. Since *luscus* can detect changes in input files, the result will be automatically displayed on the screen.

Although the primary goal of *luscus* is the fast visualisation on a screen, *luscus* is also capable to produce printing quality images of molecular systems. Many features, e.g. colour scheme, are adjustable and can be saved for a future use.

**Figure 1:**
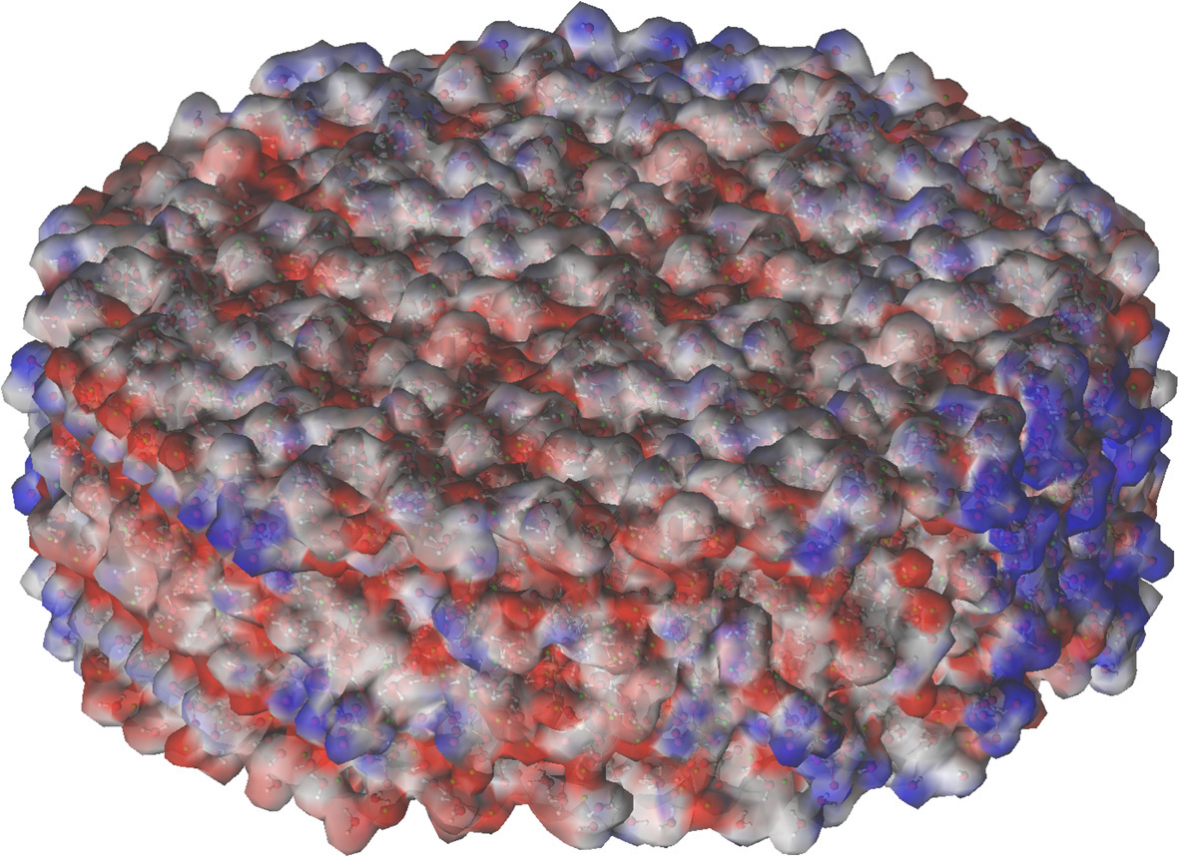
Electrostatic potential of Calcium-silicate-hydrate platelet.

All atomic data in luscus are dynamically allocated, so there is no hard limit for the number of atoms, however the practical limit depends on the computer processor and the graphic card. Some functionalities like displaying molecular orbitals can make an additional load on the computer graphical system and thus reduce the practical limits. The authors successfully created an image of a system with 9360 atoms with electron density and electrostatic potential data (see example Figure 1).

## Results and discussion

### Visualisation and editing of a chemical system

Visualisation and on screen editing of atomic coordinates is an essential part of any GUI in computational chemistry. The main concept of the editing of atomic coordinates in *luscus* is based on user selection of an atom, a bond, an angle, or a torsion angle. *Luscus* provides different functionality depending on this selection. By default all operations are performed for the whole molecule. However, if one point (an atom, or a dummy atom) is selected, all operations are performed for this centre. In the same way, if a bond or an angle is selected, it becomes the main focus for editing operation.

New molecular geometries can be build (or existent geometries can be modified) by adding one by one atom, by adding a predefined molecular fragment, or by replicating existing atoms through a symmetry operation. Predefined molecular fragments are stored in separate files and users with modest computer knowledge can easily add additional fragments or modify the existent ones.

*Luscus* can visualise structures and chemical properties, which include more than one set of Cartesian coordinates, such as trajectories, reaction coordinates and other dynamical properties. *Luscus* has the ability to read normal modes from the energy Hessian calculations and visualise vibrations in a form of animated movements. Structures from several files can be shown in the same orientation, and size, since this information can be saved and retrieved upon opening another file.

#### Building molecules in terms of internal coordinates

The most common definition of molecular geometry, Cartesian coordinates are not convenient for building and editing molecular geometries since it is hard to imagine spatial relations of atoms in terms of numerical values of Cartesian coordinates. Even if simple operations in Cartesian coordinates are applied in the graphical interface, the exact positioning of atoms in a molecule can still pose a challenge since two-dimensional image of the modelling molecule on a screen lacks the depth. Several graphical programs [4] allow movements of atoms or molecular fragments by moving a mouse pointer across the screen. This method, although intuitive, poses a difficulty of building three-dimensional structures, since the movement of atoms is limited by two dimensions only.

Defining molecular geometry in terms of internal coordinates, assembled in Z-matrix is more simple task than evaluation of Cartesian coordinates for the most of the molecules, since it involves dealing with common chemical concepts like bond lengths, angles and dihedral angles. Some most popular molecular editors [2,5,18] have Z-matrix editor built-in, where users can interactively change internal coordinates. The downside of Z-matrix editors is the limitation put by definition of molecular geometry by internal coordinates since only defined set of coordinates can be edited.

Molecular geometry in *luscus* is defined in Cartesian coordinates, however editing is performed in internal coordinates. Internal coordinates are being adjusted with partial Z-matrices that are defined *ad-hoc*, by selecting atoms and thus these coordinates are not depending on the order of atoms, as in conventional Z-matrix editors. In *luscus* it is possible to modify any interatomic distance by selecting two atoms. Relative orientations of atoms can be adjusted by changing a valence angle or a torsional angle by selecting three or four appropriate atoms. The atom that is selected first is displayed with a different colour than other selected atoms and this atom is being moved by adjusting an internal coordinate. Editing operations can be applied either to a single atom, or to a group of atoms. In order to connect several atoms into a group one can use on screen marking by mouse (we use here term marking in order to avoid a possible confusion with term select, which is used to select a focus on a structural element of the molecule), or one can use a graphical interface to determine the group (e.g. the atoms of the same chemical element, or located on one side from a selected bond, or connected via a bond to a specific atom). When a group is defined, all operations will be performed for all atoms belonging to this group.

The flexibility of editing molecular systems can be extended by introducing dummy atoms as pivot points. In that way all operations can be performed around pivot points that are not part of the molecule itself.

*Luscus* also supports editing in Cartesian coordinates through a Cartesian editor. This editor supplements the aforementioned method of editing molecules in internal coordinates.

*Luscus* can use external computational programs (called by a corresponding plug-in) to modify coordinates, for example, to perform the geometry optimisation.

#### Symmetry operations

Symmetry operations become a common feature in some graphical chemistry programs that can certainly make easy building molecular structures. Symmetry operations are usually implemented as a tool for symmetrizing already build molecular structures [5] that moves already defined atoms into appropriate positions [18]. Symmetry operations in *luscus* are implemented as a building tool, that replicates existent atoms into symmetrically equivalent positions. *Luscus* brings novel feature of performing a symmetry operation applied for the selected atoms only. That feature is very useful since many molecules have at least one symmetric part. Symmetry operations available are: inversion through a point (if one atom or dummy atom is selected), translation and a proper rotation around an axis (if two atoms are selected), and finally a reflection through a plane (if three atoms are selected). In cases where a symmetry operation can’t be realised with existing atoms (pivot points), additional dummy atoms can be temporarily introduced.

The use of symmetry can be demonstrated by the example of building naphthalene molecule from benzene. First, two hydrogen atoms are deleted, and the corresponding C-C bond is selected. Naphthalene structure is created by applying a rotation by 180 degrees. The same effect can be achieved, if a dummy atom is introduced in the middle of the C-C bond and the inversion operation around the dummy atom is applied.

In order to apply a symmetry operation, *luscus* uses a threshold in order to make a decision if two atoms are identical or not. This feature can be used for symmetrizing a molecule, or its part.

For example, if during a geometry optimisation some atoms in benzene ring move out of the plane, *luscus* can enforce the planar symmetry by selecting three atoms and applying mirror reflection.

If some atoms are copied to the location that is too close to an existing atom, *luscus* will keep only one of them (in an average position), aligning the chosen part of the molecule according the symmetry element.

In addition to applying symmetry operations, *luscus* can determine a symmetry group of a molecule.

#### Drawing geometrical objects

*Luscus* allows drawing simple geometrical objects as a convenient way of emphasising a geometrical feature or a physical property in the molecular structure. For instance arrow is implemented to illustrate the dipole moment of a molecule and is displayed by default on files that provide information about molecular dipole. Other potential uses are: illustration of atomic or molecular movement, dative bond, reaction coordinate etc. All geometrical objects can be defined through a number of selected atoms, as in the case with symmetry operations. *Luscus* can draw arrows, spheres, plains, triangles, and parallelepipeds. Defining points are not restricted to the atoms present in the molecule since a temporary dummy atoms can be introduced at any time. The geometrical objects can be combined in order to draw more complicated ones, for instance a square can be drawn from two triangles. Geometrical objects are rendered as translucent since many of them enclose a volume of space and might contain atoms inside. However, the opacity as well as colour can be adjusted.

### Visualisation of orbitals

One of the most important aspect of *luscus* is its ability to visualise molecular orbitals and electron densities. The need for such feature arose with the MOLCAS program package. The MOLCAS program package is well known for multiconfigurational computational models. In this models, it is often necessary to visually inspect orbital in order to determine its nature [9] or assign it to a appropriate orbital subspace [19]. Therefore the ability to assign orbital subspaces (frozen, inactive, RAS1, RAS2, RAS3, secondary, deleted) is built in *luscus*. This feature enables fast and easy performing of otherwise cumbersome task of setting orbitals into the orbital subspaces. In order to make orbital selection even more easier, all orbitals are listed together with their symmetry, energy and occupation numbers. In a large list of orbitals, user can temporarily hide orbitals from the list or can use filtering utility to select a subset of orbitals according to one or several criteria (orbital type, symmetry, energy, occupancy). Orbitals in *luscus* are shown as isosurface of wavefuction value [20,21] in three dimensions. If an orbital wavefunction has a negative part, two isosurfaces are shown; one for positive and another for negative value of a wavefunction. Contour value, transparency and colour of isosurfaces can be adjusted. Also all orbitals can be plotted in a separate window as a series of small displays (see section Examples for more details). That way the orbitals can be easily compared through the visual inspection. Each of orbital in a small display is fully rotatable with mouse movements as well as the orbital in the main window. In addition, an orbital can be assigned to the active space by clicking a middle mouse button on an orbital display. In addition to displaying orbitals, *luscus* can display electron density of a molecule as an isosurface in the same manner as orbitals. There is a possibility of displaying other physical quantities that are spatially distributed like orbitals and electron density.

Orbitals and electron densities are represented as surfaces that engulf molecules. These surfaces can be used for displaying electrostatic potential, by colouring these surfaces according to the value of electrostatic potential. This functionality is usually used with the display of electron density surface since the display of electron density usually resembles on the molecule surface and that way a distribution of electrostatic potential can be visualised across the entire molecule.

*Luscus* can also combine several orbital files and perform simple arithmetic operations with them. This feature is very useful for construction of spin-density or for displaying of electronic transition or charge transfer.

Structures from several files can be shown in the same orientation and size, since this information can be saved and retrieved upon opening another file.

## Examples

In this section we give some examples of scientific visualisation done by *luscus*, based on recently published or on-going research in our group.

### Using symmetry for building molecules

*Luscus* is able to use symmetry operations in construction of symmetric molecules, which we will demonstrate by building Ar’-Cr-Cr-Ar’ complex, which was studied at [22]. This complex was a subject of several studies since it contains unusually short Cr-Cr bond. While building the complex it is important to make it as symmetric as possible in order to reduce the size of the calculation. This calculation was performed within multiconfigurational complete active space (CASSCF) approach. Without usage of symmetry such calculation will be impossible to carry out.

**Figure 2:**
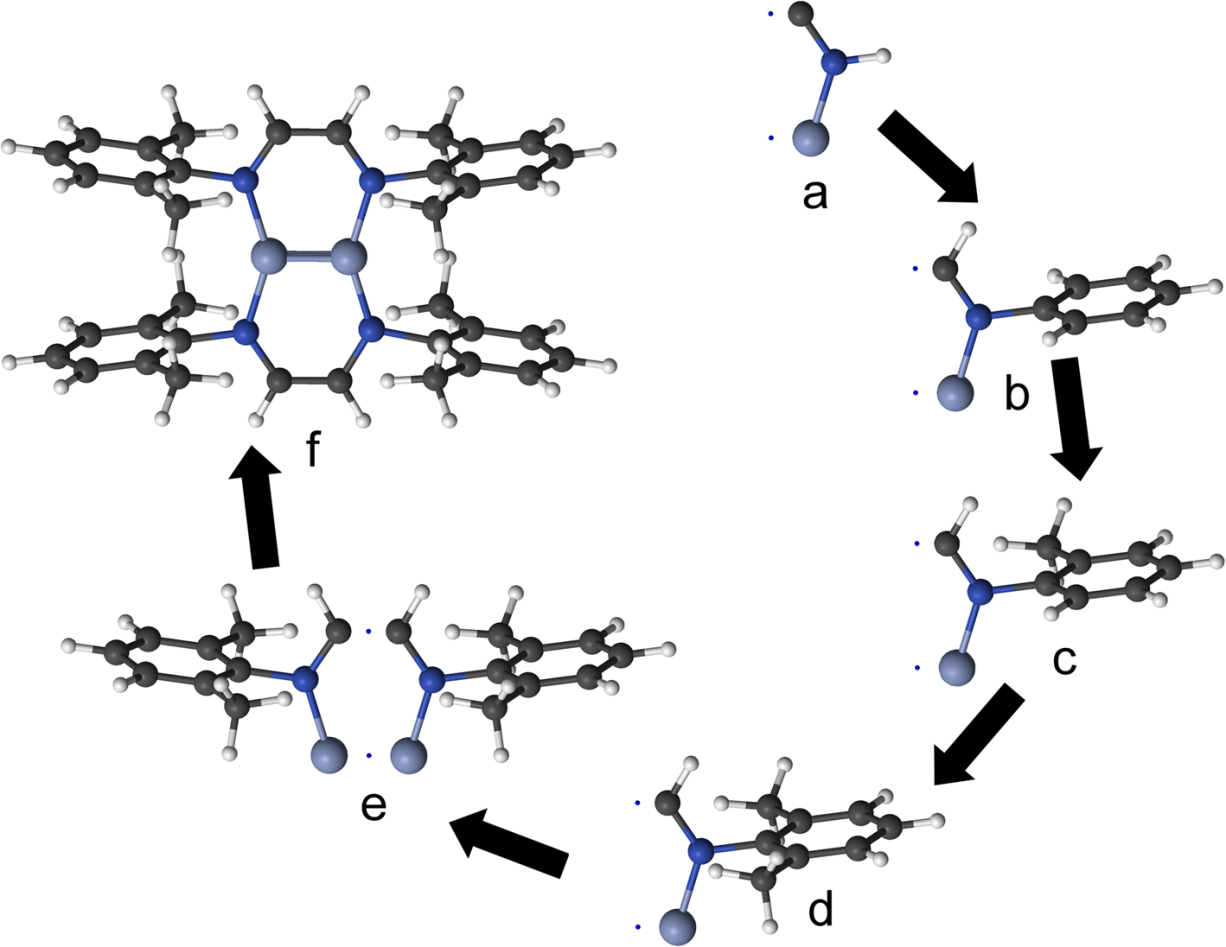
Building of Cr-Cr complex.**a)** initial fragment; **b)** hydrogen atom is replaced with phenyl fragment; **c)** hydrogen atom is replaced with methyl fragment; **d)** another hydrogen atom is replaced with methyl fragment; **e)** rotational symmetry was applied; **f)** rotational symmetry was applied again.

The construction of the complex is started with the planar fragment that contains Cr, N, C and H atoms, Figure 2(a). The fragment is build next to the dummy atoms that are being used as points of reference in order to achieve a proper orientation in space. In the next step (b), the benzene ring is added and rotated in a perpendicular orientation with respect to the initial fragment. One hydrogen atom from the benzene ring is converted into a methyl group (c). The second methyl group should have the same orientation relatively to benzene ring, which is achieved by applying a symmetry operation (reflection) only for this methyl group (d). The two-fold rotation around the dummy atoms is applied (e) followed by another two-fold rotation around the Cr-Cr bond (f).

### Building clusters and periodic structures

Molecular clusters or periodic systems require a special attention. The GUI should be able to handle translation symmetry and have capability to highlight certain structural elements. In the following example we will construct a fragment of tobermorite structure, which was used as initial structure in modelling calcium silicate (C-S-H) nanoparticles [23]. C-S-H nanoparticles are the building blocks in the formation of Portland cement, and the study of their structures can help to understand the properties of cement-based materials.

**Figure 3:**
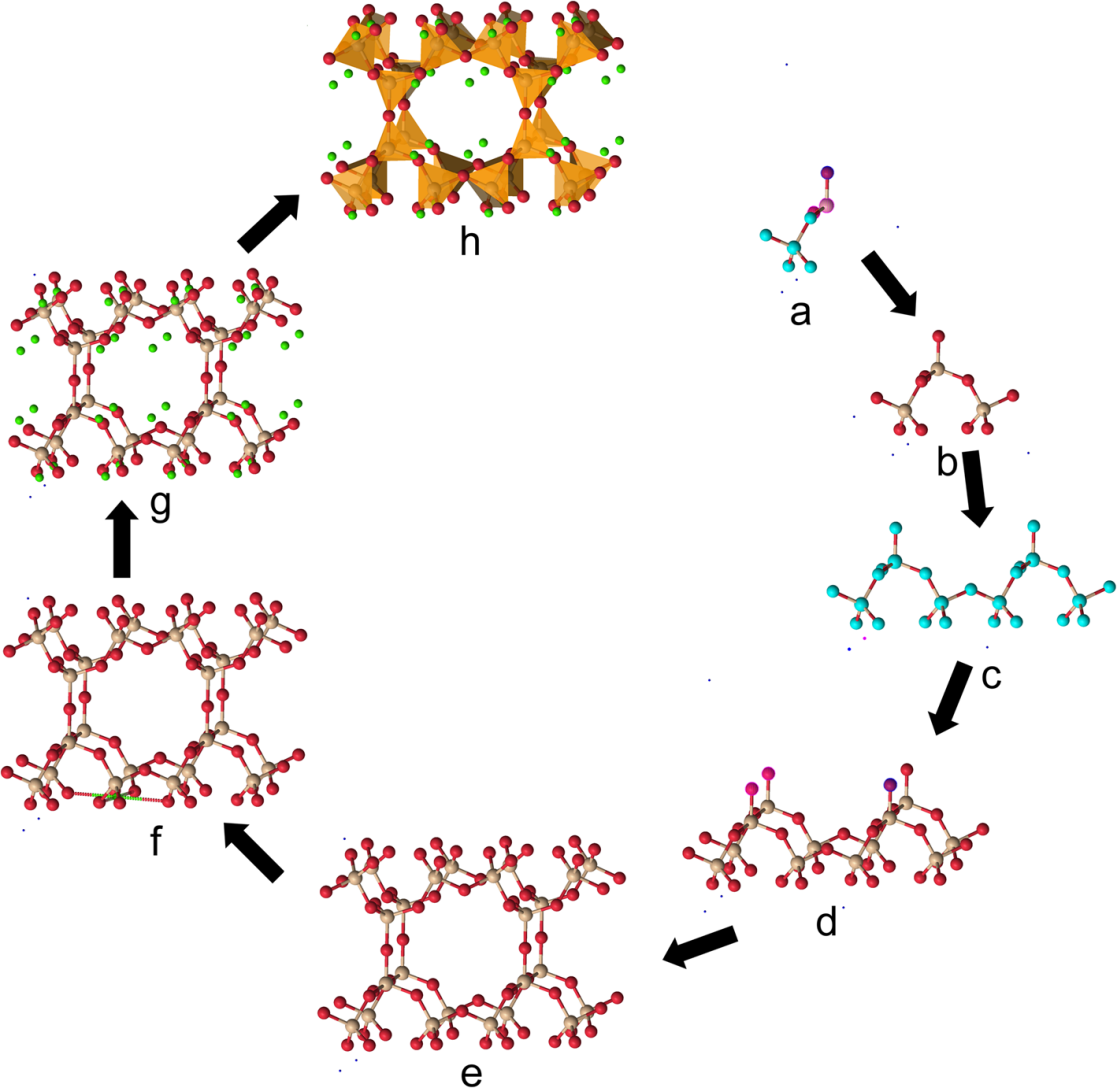
Building of crystal structure of tobermorite.**a)** initial fragment with marked and selected atoms; **b)** result of mirror reflection appyed in fragment a; **c)** result of translational symmetry operation in x direction; **d)** result of translational symmetry operation in y direction; **e)** result of mirror operation; **f)** addition of calcium atoms; **g)** addition of triangles; **(h)** final result.

The initial structural fragment includes two connected SiO _4_ tetrahedra, Figure 3(a). The third tetrahedron is obtained by mirroring of a fragment (shown in cyan in Figure 3(a)). Next, translations are used in order to create the Si-O skeleton of tobermorite. After that, calcium atoms are added and multiplied with translation operations (g). Finally, transparent tetrahedra around silicon atoms are introduced (h).

### Displaying molecular orbitals

The shape of molecular orbitals and electronic density is essential for understanding chemical boning in molecules.

**Figure 4:**
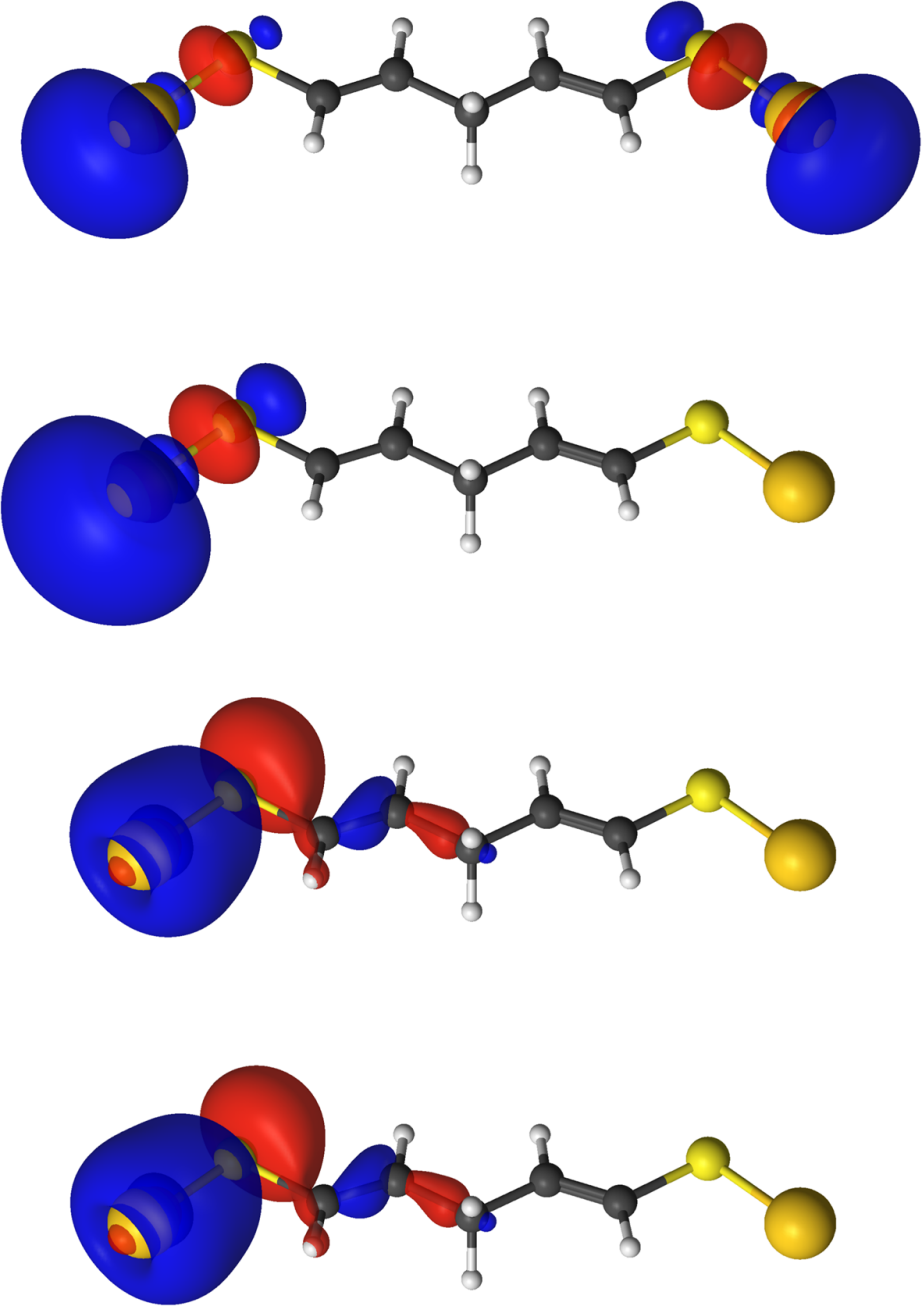
Orbitals, corresponding to the electron transport for Au-PDDT-Au.

Bi-natural orbitals, which are obtained for a pair of wave functions by a singular value decomposition of a reduced transition density matrix were recently proposed [24]. These orbitals can be used for the visual presentation of electron transport (Figure 4). The pentadienyldithiol (PDDT) is an interesting molecule in the studies of molecular conduction. By removing the terminal hydrogen atoms, the resulting dithiolate can be anchored to gold contacts. Conventional one-electron theory predicts conductivity, but calculations made by a many-electron method show that the molecule is an insulator [25]. The study of the Au-PDDT-Au molecule was performed within CASSCF method with applied electric field [24].

### Selection of active space

The selection of active space in multiconfigurational calculations is most difficult and hardly automatised procedure [26]. Visual inspection of orbitals allows to select active space based on certain characteristics of orbitals, such as the shape and localisation.

**Figure 5:**
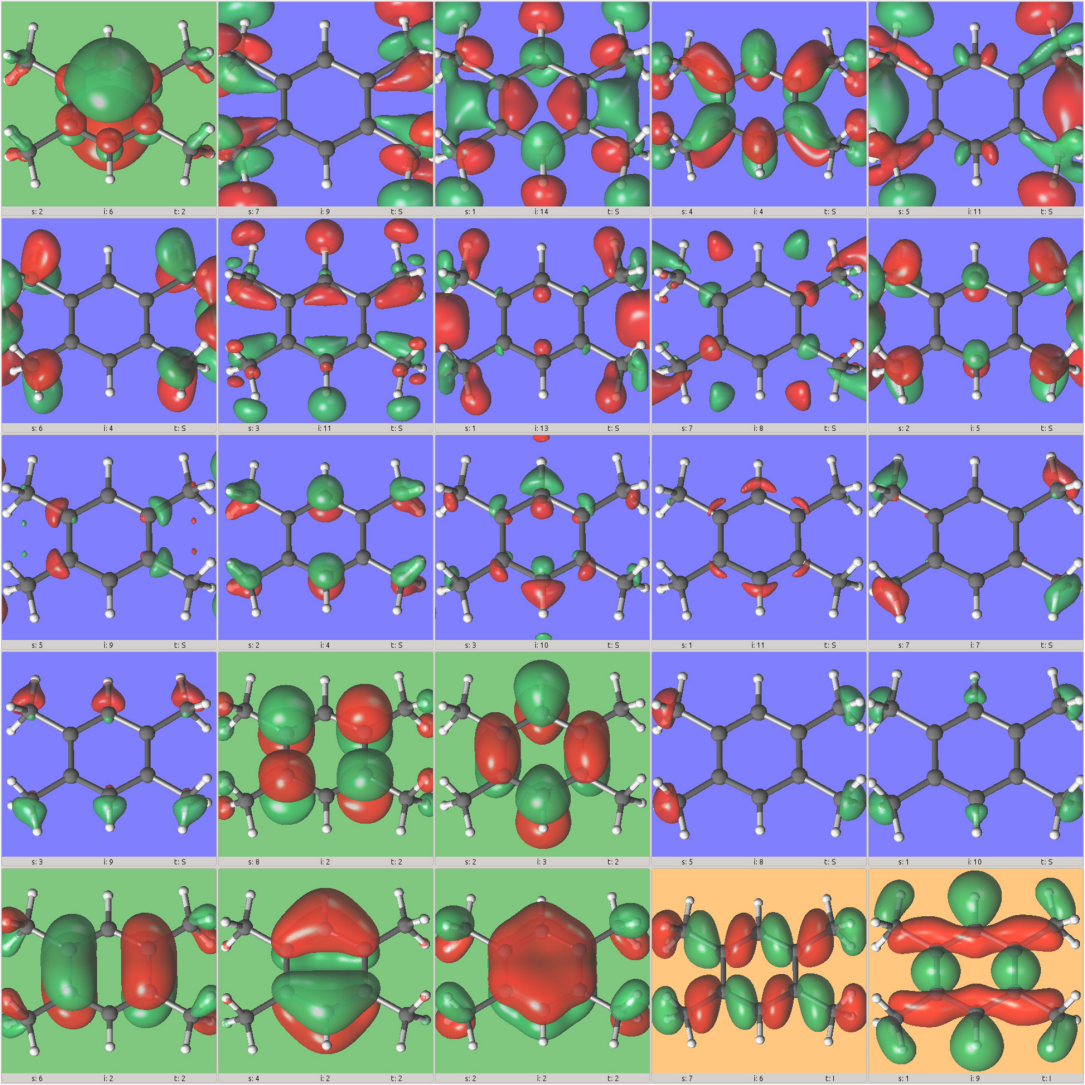
Selection of active space of the durene molecule. *π* orbitals are selected as active (green background), orbitals with yellow background are inactive and blue background indicate secondary orbitals.

For durene (1,2,4,5-tetramethylbenzene) molecule, the obvious choice of small basis set will span the six *π*-type atomic orbitals of the carbon atoms. However, these orbitals, especially virtual ones, might have one-electron energies (obtained in HF or DFT), which are far away from the HOMO-LUMO gap. Visualisation done by *luscus* allows to spot all six *π*−type orbitals (orbitals with green background in Figure 5). *Luscus* can not only help to the user to make the selection of orbital subspaces, but also create an input, which can be understood by computational code (RASSCF module in MOLCAS) directly.

### Electrostatic potential

Electrostatic potential is demonstrated on the example of calcium-silicate-hydrate (C-S-H) platelet. C-S-H is a phase that forms during the crystallisation of Portland cement and is responsible for hardening of concrete and thus for its mechanical properties. It crystallises in form of small crystallises, 30–60 nm in diameter and 5–10 nm in height [27,28]. Interaction between C-S-H platelets were extensively studied with course grained models. Since charge distribution of C-S-H platelets is unknown and experimental measurements provide only indicative information, charges used in course-grained models are largely approximate. More realistic charge distribution in a C-S-H platelet can be obtained by calculating atomic charges in a C-S-H. In Figure 1, a charge distribution of a modelled C-S-H crystallite is shown. The charge is calculated with ReaxFF _*SiO*_ force field, by electron equilibration method for each atom in the model. The electrostatic potential is calculated from this point charges and projected onto the crystalline surface.

### Dipole moments of water in a droplet

**Figure 6:**
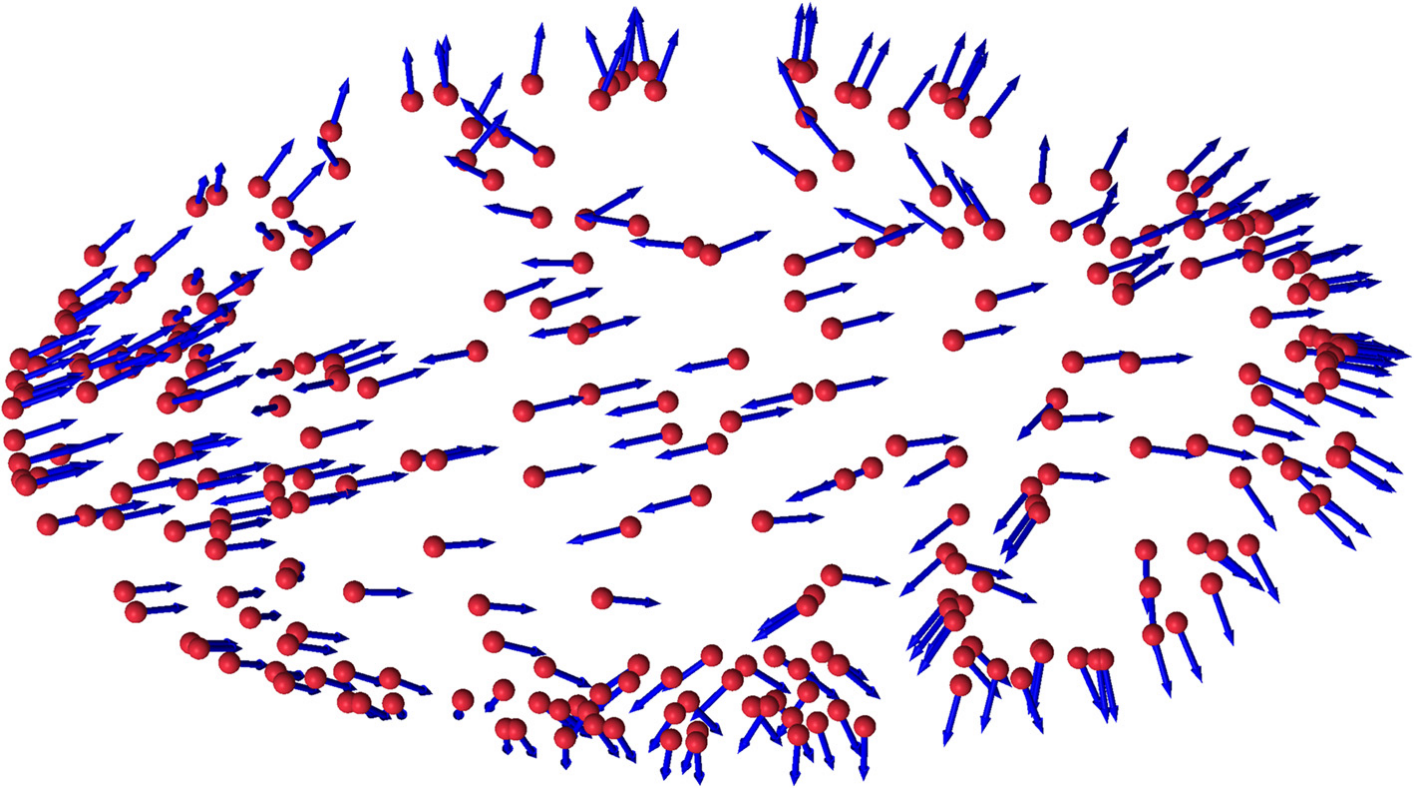
A snapshot of the distribution of dipole moments in water droplet.

Simulation of liquids requires statistical mechanical approach. The simulation of water droplet (Figure 6) has been performed with Monte Carlo based software Faunus [29] using a Stockmayer potential. This potential is a simple but relatively accurate model of ferrofluids in general but more specifically used as a representation of water. The stockmayer-potential often catches the phase behaviour of ferrofluids and of course the anisotropic behaviour of any dipolar system.

## Conclusions

*Luscus* is a new graphical user interface program, which works as a native counterpart to computational code MOLCAS, but in the same time can be easily connected to other computational codes using external plug-ins. The code is fully portable to various operating systems, and it depends only on a minimal set of external libraries. The main purpose of *luscus* is to provide state of the art support for advanced computations in quantum chemistry.

## Availability and requirements

**Project name:** luscus**Project home page:**http://sourceforge.net/projects/luscus/**Operating system(s):** Linux, Windows, Mac OS**Programming language:** C**Other requirements:** gtk+-2.0 or gtk+-3.0, OpenGL**License:** Academic free license**Any restrictions** to use by non-academics: NO

## Additional files

Additional file 1
**Example of** ***luscus*** **file format.** This example demonstrates luscus file format. The header of luscus files are identical with XYZ file format (cartesian coordinates of 17 atoms in the example). The <ATOM> section defines alternative name and numeration for each atom as well as the colour. Section <BONDS> defines bonding between atoms. Keyword AUTOMATIC=0 instructs luscus not to search bonding atoms according to interatomic distance, but to read the data from the <BONDS> section. Sections <VECTOR>, <TRIANGLE> and <SPHERE> define geometrical objects. Colour, transparency and the coordinates in these sections. Section<TEXTBOX> defines the text written on the screen.

Additional file 2
**Example of bash script as a** ***luscus*** **plug-in.** This example demonstrates using a simple bash script as a plug-in for conversion a PDB file into a luscus file. In this example, the conversion is done with the OpenBabel program. Since minimal luscus file consists of a header that is identical to the XYZ file format, the PDB is converted to XYZ file which is used as a luscus file.
