# Supplementary material for: Luscus: molecular viewer and editor for MOLCAS
Source: J Cheminform. 2015 Apr 29;7:16. doi: 10.1186/s13321-015-0060-z (PMC4432095; doi:10.1186/s13321-015-0060-z)
Supplement: Additional file 1 — Example of luscus file format. This example demonstrates luscus file format. The header of luscus files are identical with XYZ file format (cartesian coordinates of 17 atoms in the example). The section defines alternative name and numeration for each atom as well as the colour. Section defines bonding between atoms. Keyword AUTOMATIC=0 instructs luscus not to search bonding atoms according to interatomic distance, but to read the data from the section. Sections , and define geometrical objects. Colour, transparency and the coordinates in these sections. Section defines the text written on the screen. [file 13321_2015_60_MOESM1_ESM.pdf]

This example demonstrates luscus file format. The header of luscus files are identical with XYZ file format (cartesian coordinates of the 17 atoms in the examples). The <*ATOM*> section defines alternative name and numeration for each atom as well as the colour. Section <*BONDS*> defines bonding between atoms. Keyword *AUTOMATIC=0* instructs *luscus* not to search bonding atoms according to interatomic distance, but to read the data from the <*BONDS*> section. Sections <*VECTOR*>, <*TRIANGLE*> and <*/SPHERE*> define geometrical objects. Colour, transparency and the coordinates in these sections. Section<*TEXTBOX*> defines the text written on the screen.

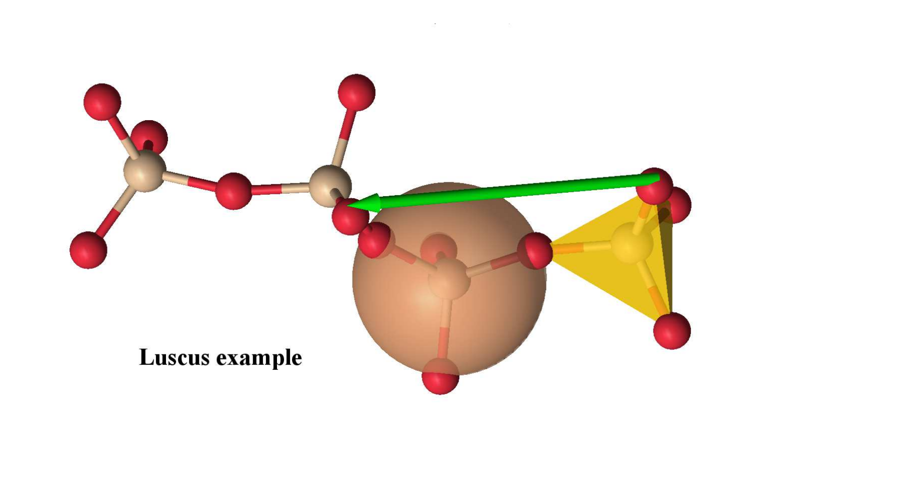


```
17
File generated by luscus version 0.8.1 beta
O 0.000000 0.000000 -0.570053
Si 0.000000 0.000000 -2.180053
O -0.000000 1.504190 -2.754084
O 1.302667 -0.752095 -2.754084
O -1.302667 -0.752095 -2.754084
Si 0.463100 0.297963 0.942914
O 0.585884 1.881154 1.204934
O 1.894884 -0.371057 1.247724
O -0.599763 -0.299840 1.995675
Si -1.875048 -1.096673 2.570831
O -3.247437 -0.387827 2.116754
O -1.895567 -2.613328 2.030982
O -1.846216 -1.141175 4.179958
Si -1.507472 -1.636604 5.673913
O -0.065922 -2.351478 5.728602
O -1.487181 -0.395659 6.699457
O -2.606983 -2.692600 6.191647
<ATOM>
NAME = A NUMBER = 1 RED=0.953000 GREEN=0.180000 BLUE=0.259000
NAME = A NUMBER = 1 RED=0.941000 GREEN=0.784000 BLUE=0.627000
NAME = A NUMBER = 2 RED=0.953000 GREEN=0.180000 BLUE=0.259000
NAME = A NUMBER = 3 RED=0.953000 GREEN=0.180000 BLUE=0.259000
NAME = A NUMBER = 4 RED=0.953000 GREEN=0.180000 BLUE=0.259000
NAME = B NUMBER = 2 RED=0.941000 GREEN=0.784000 BLUE=0.627000
NAME = B NUMBER = 5 RED=0.953000 GREEN=0.180000 BLUE=0.259000
NAME = B NUMBER = 6 RED=0.953000 GREEN=0.180000 BLUE=0.259000
NAME = B NUMBER = 7 RED=0.953000 GREEN=0.180000 BLUE=0.259000
NAME = C NUMBER = 3 RED=0.941000 GREEN=0.784000 BLUE=0.627000
NAME = C NUMBER = 8 RED=0.953000 GREEN=0.180000 BLUE=0.259000
NAME = C NUMBER = 9 RED=0.953000 GREEN=0.180000 BLUE=0.259000
NAME = C NUMBER = 10 RED=0.953000 GREEN=0.180000 BLUE=0.259000
NAME = D NUMBER = 4 RED=0.941000 GREEN=0.784000 BLUE=0.627000
NAME = D NUMBER = 11 RED=0.953000 GREEN=0.180000 BLUE=0.259000
NAME = D NUMBER = 12 RED=0.953000 GREEN=0.180000 BLUE=0.259000
```

```
NAME = D NUMBER = 13 RED=0.953000 GREEN=0.180000 BLUE=0.259000
</ATOM>
<BOND>
AUTOMATIC = 0
2 1 1
2 3 1
2 4 1
2 5 1
6 7 1
6 8 1
6 9 1
1 6 1
10 11 1
10 12 1
10 13 1
9 10 1
14 15 1
14 16 1
14 17 1
13 14 1
</BOND>
<VECTOR>
RED = 0.000000 GREEN = 1.000000 BLUE = 0.061494 TRANSPARENCY = 1.000000 RADIUS = 0.100000
SHARPNESS = 0.100000
-1.502667 -0.850950 -2.840840
-3.847437 -0.500000 2.116754
</VECTOR>
<TRIANGLE>
RED = 1.000000 GREEN = 0.783490 BLUE = 0.000000 TRANSPARENCY = 0.700000
0.000000 0.000000 -0.570053
-1.302667 -0.752095 -2.754084
1.302667 -0.752095 -2.754084
</TRIANGLE>
<TRIANGLE>
RED = 1.000000 GREEN = 0.784314 BLUE = 0.000000 TRANSPARENCY = 0.700000
0.000000 0.000000 -0.570053
1.302667 -0.752095 -2.754084
0.000000 1.504190 -2.754084
</TRIANGLE>
<TRIANGLE>
RED = 1.000000 GREEN = 0.784314 BLUE = 0.000000 TRANSPARENCY = 0.700000
-1.302667 -0.752095 -2.754084
1.302667 -0.752095 -2.754084
0.000000 1.504190 -2.754084
</TRIANGLE>
<TRIANGLE>
RED = 1.000000 GREEN = 0.784314 BLUE = 0.000000 TRANSPARENCY = 0.700000
0.000000 0.000000 -0.570053
0.000000 1.504190 -2.754084
-1.302667 -0.752095 -2.754084
</TRIANGLE>
<SPHERE>
RED = 1.000000 GREEN = 0.672984 BLUE = 0.465110 TRANSPARENCY = 0.700000 RADIUS = 1.609504
0.463100 0.297963 0.942914
</SPHERE>
<TEXTBOX>
RED = 0.000000 GREEN = 0.000000 BLUE = 0.000000 X=196 Y=662
Times New Roman, Bold 24
Luscus example
</TEXTBOX>
<END>
```
