# Supplementary material for: Luscus: molecular viewer and editor for MOLCAS
Source: J Cheminform. 2015 Apr 29;7:16. doi: 10.1186/s13321-015-0060-z (PMC4432095; doi:10.1186/s13321-015-0060-z)
Supplement: Additional file 2 — Example of bash script as a luscus plug-in. This example demonstrates using a simple bash script as a plug-in for conversion a PDB file into a luscus file. In this example, the conversion is done with the OpenBabel program. Since minimal luscus file consists of a header that is identical to the XYZ file format, the PDB is converted to XYZ file which is used as a luscus file. [file 13321_2015_60_MOESM2_ESM.pdf]

This example demonstrates using a simple bash script as a plug-in for conversion a PDB file into a luscus file. In this example, the conversion is done with the OpenBabel program. Since minimal luscus file consists of a header that is identical to the XYZ file format, the PDB is converted to XYZ file which is used as a luscus file.

```
#!/bin/bash
babel -i pdb $1 -o xyz ${1%.*}.xyz #convert PDB file into a XYZ file
mv -f ${1%.*}.xyz ${1%.*}.lus #rename XYZ file into a luscus file
```
